# Clinical Features of Hepatitis C Virus Carriers With Persistently Normal Alanine Aminotransferase Levels

**DOI:** 10.5812/hepatmon.829

**Published:** 2012-02-29

**Authors:** Hirofumi Uto, Seiich Mawatari, Kotaro Kumagai, Akio Ido, Hirohito Tsubouchi

**Affiliations:** 1 Department of Human and Environmental Sciences, Graduate School of Medical and Dental Sciences, Kagoshima University, Kagoshima, Japan

**Keywords:** Hepatitis C, Alanine Transaminase, Clinical Protocols, Liver Cirrhosis

## Abstract

Hepatitis C virus (HCV) infection causes chronic hepatitis, which frequently leads to hepatic fibrosis and hepatocellular carcinoma (HCC). Alanine aminotransferase (ALT) is a biomarker of hepatocyte injury and is associated with the progression of hepatic fibrosis. Advanced hepatic fibrosis also predisposes HCV carriers to a risk of HCC. In contrast, some cases with persistent HCV infection have normal ALT levels that persist for a long time, and these HCV carriers have no or mild hepatitis and hepatic fibrosis. These HCV carriers are defined as persistent normal ALT (PNALT) cases and their risk of HCC is low compared to HCV carriers with abnormal ALT. However, there are various definitions of normal ALT and PNALT, and advanced hepatic fibrosis may be missed without a liver biopsy. In addition, there is also a risk of ALT elevation in HCV carriers with PNALT, which increases the risk of progression to hepatic fibrosis and HCC. Most HCV carriers with PNALT have asymptomatic or nonspecific symptoms. HCV carriers with PNALT are also considered to be responsive to interferon-based treatment. Thus, assessment of hepatic fibrosis is important in HCV carriers, and the eradication of HCV infection is more likely in HCV carriers with evidence of hepatic fibrosis, regardless of their ALT levels.

## 1. Context

Hepatitis C virus (HCV) causes chronic hepatitis, liver cirrhosis, and hepatocellular carcinoma (HCC) at high rates [1][2][3]. However, some cases remain asymptomatic with normal levels of alanine aminotransferase (ALT) after HCV infection and detection of the infection in these cases may only occur through screening, such as with an anti-HCV antibody test. The ALT level usually rises in hepatitis [4], but it is normal in approximately 20-30% of HCV carriers [5][6]. Previous studies have shown that elevated ALT levels predict an increased rate of HCV associated HCC in a community-based population and that serial measurements to identify persistent ALT abnormality may be useful in determining the HCC risk [7][8]. Thus, HCV carriers with normal ALT levels were previously not indicated for antiviral therapy. In contrast, it has been shown that hepatic inflammation and fibrosis are histologically present in many HCV carriers, even though their ALT level is normal, and these HCV carriers are candidates for antiviral therapy. These conflicting issues may arise from ambiguity around the definition of normal ALT and with the natural course of HCV carriers with persistent normal ALT (PNALT). In this review, which is based on a search for articles using terms such as persistent ALT, normal ALT, and HCV, we describe the characteristics, natural course, and treatment in HCV carriers with PNALT.

## 2. Evidence Acquisition

### 2.1. Epidemiologic Features of HCV Carriers

Many HCV carriers are asymptomatic. According to American Association for the Study of Liver Diseases (AASLD) practice guidelines, drug users, patients with a high risk for HCV infection (patients with HIV infection or hemophilia treated with coagulation factor preparations before 1987, and patients with hemodialysis or ALT abnormality), patients who received blood transfusion or organ transplantation before July 1992, children born to HCV-infected mothers, medical workers exposed to needles used for HCV-infected patients or to the mucosa of HCV-infected patients, and people who have had a sexual relationship with an HCV carrier should be screened for a HCV infection [9]. More than 170 million people worldwide are thought to be infected with the HCV. HCV infection progresses to a chronic state in 60-85% of infected people and may develop into liver cirrhosis and HCC after 20-35 years [3][10]. Poynard et al. reported that 33% of patients with a HCV infection had an expected median time to cirrhosis of less than 20 years without treatment [11]. Other studies have also shown a rate of progression to cirrhosis of between 1.5 and 16 years following blood transfusion in approximately 20% of cases [12]. The rate of progression to HCC was reported to be approximately 2% per year [13][14]. In addition, about 1.4 million people (approximately 1% of HCV carriers) are estimated to die annually due to liver cirrhosis or HCC [1][3]. We previously reported that 70 out of 758 HCV carriers died due to a liver-related cause over an average of 8.2 years of follow up (a rate of 1% per year) [8]. The important point is that those rates may be mainly due to the diversity in the design of the studies themselves. Host factors and environmental factors affect the progression of hepatic inflammation and fibrosis. Host factors include; advanced age at the time of infection, male gender, excess alcohol consumption, excess iron intake, cigarette smoking, obesity, complications with diabetes, fatty liver and metabolic syndrome, and coinfection with human immunodeficiency virus (HIV), hepatitis B virus (HBV), and Schistosomiasis [3][15]. In addition, hepatic fibrosis in liver grafts shows rapid progression after liver transplantation in hepatitis C patients [16][17], although the mechanism for this remains unclear. In contrast, the progression of hepatic fibrosis may not be associated with HCV genotype or viral load [11][18].

### 2.2. The Concept of ALT Normality and Definition for PNALT

Since ALT is released into the blood when the hepatocyte membrane is impaired or hepatocytes are destroyed, elevation of the serum ALT level is a useful marker indicating hepatocellular impairment [15]. According to Up ToDate ver. 19.3 (UpToDate, Inc. Waltham, MA), HCV infection with a normal ALT level is defined by the following [5]; detectable HCV RNA and serum ALT concentration that is persistently within the normal range. The definition of PNALT includes the idea of "persistence", indicating maintenance of an ALT level that is below the upper limit of the normal value over a long period of time (e.g., over a six-month period). However, this definition has varied among the many reports on PNALT, which have used different periods of observation and upper limits of normal ALT that are often set at the highest value of the measurement instrument. Moreover, the ALT level varies depending on; age, gender, race, and body mass index (BMI) [9]. Prati et al. investigated 6,835 blood donors with ALT levels within the standard range (male: ≤ 40 IU/l, female: ≤ 30 IU/l) who were negative for infections (HBV, HCV, and HIV), had no history of medication, with blood glucose, cholesterol, and triglyceride levels lower than the upper limits of the standard ranges, and had a BMI < 25 kg/m2. The findings showed that 30 and 19 IU/l were appropriate as the upper limits of the normal ALT range in males and females, respectively [20]. However, according to the AASLD Practice guidelines, PNALT is present when ALT is measured 2-3 times over 1-6 months and all values are < 40 IU/l [9]. Thus, the standard ALT level is unclear and the criteria for PNALT vary among the different reports. Therefore, it is important to pay attention to the definition of a normal ALT level in order to evaluate the natural course of HCV carriers with PNALT.

### 2.3. Clinical Manifestations in HCV Carriers With PNALT

Most HCV carriers with PNALT are discovered incidentally when anti-HCV antibodies are found following a blood donation. Most of these patients have asymptomatic disease or nonspecific symptoms including; fatigue, weakness, and upper right quadrant pain [20]. The frequency and severity of these symptoms have been reported to be similar among anti-HCV positive patients with normal serum ALT, mildly elevated values (< 2 times normal), and more marked elevation (> 2 times normal) [21]. In contrast, Jamal et al. have suggested that there is a trend towards depression being more common in HCV carriers with abnormal ALT compared to those with normal ALT, although they found that there was no statistically significant difference in depressive symptoms [20]. Therefore, depression, though nonspecific, might be an important clinical marker of a more severe disease.

### 2.4. Epidemiology, Clinical Course and Management in HCV Carriers With PNALT

The ALT level is within the normal range in about 30% of HCV carriers and lower than 2-fold the normal upper limit in 40% [5][6], although what constitutes a normal ALT value is not completely clear. The frequency of PNALT is reported to be higher in HCV genotype 2 carriers and females [6][22]. Inflammation and fibrosis were shown to be histologically mild in approximately 70% of PNALT cases [23]; however, fibrosis of stage 2 (F2) or more severe fibrosis was noted in 22% of histologically evaluated PNALT cases in another report [22]. Therefore, severe fibrosis can be present in some cases in which their ALT level is normal. This may be because ALT levels occasionally normalize in patients with liver cirrhosis. The rate of progression of hepatic fibrosis in patients with chronic hepatitis C is reported to be 0.1-0.13 units/year [11][24]. PNALT cases have been found to show only a slight histological progression over a 10-year follow-up period [25], with a reported rate of progression of fibrosis of 0.05 units per year [26][27]. These results suggest that fibrosis progresses more slowly in PNALT cases. During the long-term follow-up of patients with PNALT, ALT levels rose in 21.5% of cases 3-18 months after PNALT was judged to be present [6]. Platelet count is thought to be a simple marker for hepatic fibrosis, and PNALT patients with severe fibrosis can be identified using the criterion of a platelet count ≤ 150,000/μL. In patients selected using this criterion and an ALT level of ≤ 30 IU/L for 5 years, Okanoue et al. showed that ALT ≤ 30 IU/L was maintained in only 14% of patients [28]. In our study, 101 HCV carriers with PNALT (defined as ALT ≤ 34 IU/l) were surveyed between 1993 and 2000, and ALT levels rose in 31.8% of these patients over a 5-year observation period [29]. Thus, there is a risk of ALT elevation in HCV carriers with PNALT, even if the ALT level has been continuously normal over several years and if the definition of PNALT includes the platelet count, in addition to the ALT level. Furthermore, we found that an ALT level of 20-34 IU/l [odds ratio (OR): 5.6], a serum ferritin level of ≥ 90 ng/ml (OR: 3.1), and a minor allele of the HFE gene (H63D, OR: 4.8) were independent risk factors for ALT elevation [29]. Shiffman et al. found that the ALT level was maintained at about 20 IU/L in PNALT cases [30]. Therefore, when the ALT level is ≤ 19 IU/L, it is unlikely to rise, and this suggests that 19 IU/L may be an appropriate upper limit for ALT in the definition of PNALT.

### 2.5. ALT levels in HCV Carriers with End Stage Renal Failure or HIV Coinfection

The population of patients with fibrosis progression, despite having ALT levels within the normal range, typically includes dialysis patients with HCV infection and HCV carriers with HIV coinfection. Dialysis patients are a high-risk group for HCV infection, with an HCV prevalence of about 13.5% among these patients [31]. Dialysis patients also generally have a low ALT level, and we also found low mean ALT levels of 13.2 and 18.5 IU/l in 238 HCV-negative and HCV-positive dialysis patients [32]. A comparison of ALT levels with histologically severe inflammation and fibrosis showed that the ALT levels were lower in patients with end-stage renal failure who were under dialysis, compared to patients with normal renal function [33]. However, the cause of the low ALT level in end-stage renal failure patients is unclear. Among HIV-infected patients, 25% are reported to be infected with HCV [34], and the incidence of fibrosis progression in PNALT cases is nearly 2 times higher in patients with HIV-HCV coinfection than in those with a HCV infection alone [35]. Thus, although histological progression is generally mild in HCV carriers with PNALT, hepatic fibrosis is more progressive in those patients with end-stage renal failure or an HIV coinfection.

### 2.6. Immunopathogenesis of HCV Carriers With PNALT

The mechanism for ALT elevation in HCV carriers is not yet fully elucidated, but may be associated with functional abnormalities of the immune cells, such as activated lymphocytes and NK cells in patients with chronic hepatitis C [36][37]. Dendritic cells (DCs) play a central role in the activation of immunocytes, and the numbers of myeloid DCs (mDCs) and plasmacytaid DCs (pDCs) are smaller in patients with chronic hepatitis C than in subjects without a HCV infection. A comparison of PNALT cases and chronic hepatitis C patients showed no difference in the number of mDCs or pDCs, but a reduced DC function was associated with a higher ALT level [38]. In contrast, many immunosuppressive regulatory T cells (Treg) are present in HCV carriers and the inhibitory activity is stronger in PNALT cases than in patients with active hepatitis [39]. These results indicate that therapy to regulate the functional abnormalities of immune cells may be valuable in patients with chronic hepatitis C in order to reduce the ALT level or hepatic inflammation. In addition, the frequency of DR13 in HCV-infected patients with a normal ALT level was significantly higher than that of HCV-infected patients with elevated ALT (42% vs. 4%, Pc < 0.003) [40]. Thus, immunological analysis in HCV carriers with PNALT may lead to new therapies for patients with chronic hepatitis C.

### 2.7. Noninvasive Evaluation of Fibrosis in HCV Carriers With PNALT

In HCV carriers, the risk of HCC increases with the progression of hepatic fibrosis [41]. Liver biopsy is the standard test for hepatic fibrosis, but it is invasive and causes complications at a rate of 1-3% and has a mortality rate of 1/10000-12000 [42]. Moreover, sampling errors leading to the underestimation of liver cirrhosis have been reported in 14.5% of cases [43]. Noninvasive tests for the evaluation of fibrosis have also been described, these include; combinations of peripheral blood and serum chemistry tests [44][45][46][47][48] and fibrosis markers [49][50] (Table 1); elastography using transient elastography (TE FibroScan®) and acoustic radiation force impulse (ARFI) [51][52][53][54][55][56][57] (Table 2). Accuracy increases when several tests are used in combination, but the utility of these tests for the evaluation of hepatic fibrosis in PNALT cases remains uncertain.

**Table 1:** Diagnostic Accuracy of the Models for Predicting Liver Fibrosis Using Examination of Peripheral Blood and Serum Chemistry

**Model Name (Reference)**	**Variables**	**Etiology **	**Fibrosis Stage**	**Cut off Values **	**AUROC **a
APRI a [44]	AST a, Plt a	HCV	Ishak score 0-2	< 0.5	0.80-0.88
			Ishak score 3-6	≥ 1.5	
FIB-4 [45]	Age, AST, Plt, ALT a	HCV/HIV	Ishak score 0-3	< 1.45	0.765
			Ishak score 4-6	≥ 3.25	
Forns Index [46]	Plt, GGT a, age, cholesterol	HCV	F0-1	> 4.2	0.81-0.86
			F2-4	> 6.9	
Fibro Index [47]	Plt, AST, γ globulin	HCV	F0-1	≤ 1.25	0.83
			F2-3	≥ 2.25	
FibroTest [48]	α2-MC a, α2 globulin (or haptoglobin), γ-globulin, apolipoprotein A1, GGT, Total bilirubin	HCV	F0-1	0-0.1	0.836-0.870
			F2-4	0.6-1.0	
SHASTA Index [49]	HA a, AST, albumin	HCV/HIV	Ishak score 0-2	< 0.3	0.878
			Ishak score 3-6	> 0.8	
Hepascore [50]	HA, α2-MC, GGT, age, gender	HCV	F0-2	< 0.5	0.82-0.90
			F3-4	≥ 0.5	

^a^ Abbreviations: α2-MC, α2-macroglobulin; ALT, alanine aminotransferase; AUROC, area under receiver operating characteristic curve; AST, aspartate aminotransferase APRI, AST-platelet ratio index; GGT, gamma-glutamyltransferase;HA, hyaluronic acid; Plt, platelet

**Table 2:** Diagnostic Accuracy for Predicting Liver Fibrosis Using Transient Elastography or Acoustic Radiation Force Impulse

**Technique**	**(Reference)**	**Etiology **	**Fibrosis Stage**	**Cut off Values**	**AUROC **a
TE a	[51]	HCV a	F ≥ 2	8.7	0.79
			F ≥ 3	9.56	0.91
			F = 4	14.52	0.99
	[52]	HCV	F ≥ 2	7.1	0.88
			F ≥ 3	9.5	0.95
			F = 4	12.5	0.95
	[53]	CLD a	F = 4	14.6	0.95
	[54]	HCV	F ≥ 2	7.1	0.88
			F ≥ 3	9.6	0.90
			F = 4	11.6-16.9	0.90
	[55]	HCV	F ≥ 1	5.2	0.902
			F ≥ 2	8.1	0.941
			F ≥ 3	9.6	0.926
			F = 4	13.1	0.945
ARFI a	[56]	HCV	F ≥ 2	1.215	0.902
			F ≥ 3	1.54	0.993
			F = 4	1.94	0.993
	[57]	CLD a	F ≥ 2	1.34	0.94
			F ≥ 3	1.44	0.94
			F = 4	1.80	0.96
	[55]	HCV	F ≥ 1	1.19	0.709
			F ≥ 2	1.34	0.851
			F ≥ 3	1.61	0.869
			F = 4	2.00	0.911

^a^ Abbreviations: ARFI, acoustic radiation force impulse; AUROC, area under receiver operating characteristic curve; CLD, chronic liver disease; HCV Hepatitis C virus; TE, transient elastography

### 2.8. PNALT and the Risk for HCC in HCV Carriers

Tanaka et al. investigated HCC development in 1,927 HCV antibody-positive blood donors and found that the incidence of HCC was lower in subjects with a low ALT level compared to those with a high ALT level [58]. In our study, subjects were followed for an average of 8 years before HCC development, and a strong association between the ALT level and HCC development was found, with a significant association between a 20 IU/L higher ALT level and the subsequent incidence of HCC being observed [hazard ratio (HR) = 1.2] [7]. The HCC risk was also much lower in the PNALT cases than in subjects with a persistent abnormal ALT level. Among 551 subjects with at least 4 repeated measurements of ALT, those with persistently abnormal ALT levels (n = 118) had a significantly increased rate of HCC compared to those with persistently normal ALT levels (n = 296) (HR = 23.2) [7]. Kumada et al. also found that a high ALT level and low platelet count were strongly associated with HCC development [59]. When a low ALT level persists for a prolonged period and the platelet count does not decrease, the HCC complication rate may be very low despite the persistence of a HCV infection. Collectively, these results show that the risk for HCC in subjects with PNALT is low among HCV carriers, including patients with chronic hepatitis and liver cirrhosis.

### 2.9. Insulin Resistance and Fatty Liver in HCV Carriers

In liver cirrhosis, insulin sensitivity decreases in peripheral tissue, for which pancreatic β cells compensate through the secretion of excess insulin, inducing hyperinsulinemia. Allison et al. found that the rate of diabetes complications was higher in liver cirrhosis associated with a HCV infection, than in that induced by other causes [60]. The homeostasis model assessment-insulin resistance (HOMA-IR) value, which is an index of insulin resistance, was found to be significantly higher in stage 0 or 1 chronic hepatitis C patients with mild hepatic fibrosis than in healthy subjects, and HOMA-IR served as a predictor of the progression of hepatic fibrosis [61]. Animal studies also suggest that the HCV core protein acts on the insulin signal transmission pathway and induces insulin resistance [62]. However, it is unclear whether insulin resistance is present in PNALT cases without hepatic fibrosis. A high rate of fat deposition in the liver is caused by HCV infection [63], and fatty changes in the liver and insulin resistance are induced in transgenic mice expressing the HCV core protein [64]. Castera et al. performed a liver biopsy at mean intervals of 48 months and observed that; male gender, histological stage, and the presence of advanced fatty changes were significant risk factors in promoting hepatic fibrosis, and that fatty change in the liver was an independent risk factor in multivariate analysis [65]. Fatty change in the liver is particularly marked with genotype 3 viruses [66], and fatty change in the liver and insulin resistance have also been associated with the negative effects of interferon (IFN)-based therapy [67]. Thus, insulin resistance and fat deposition in the liver, which are associated with hepatic fibrosis, should be less severe in PNALT cases compared to HCV patients with abnormal ALT, and this may produce a more favorable outcome for IFN-based treatment. However, the antiviral effect of IFN-based therapy in patients with normal ALT is comparable to that for patients with abnormal ALT, regardless of their background advantages [68][69]. Therefore, further studies of insulin resistance and fat deposition in the liver are needed in PNALT cases.

## 3. Results

### 3.1. Improved Outcomes With Antiviral Treatment for HCV Carriers

Treatment for HCV carriers has improved markedly in recent years. Combination therapy with pegylated (PEG) interferon (IFN) plus ribavirin achieves a sustained viral response (SVR) in approximately 50% of the most intractable genotype 1 patients and high viral load cases. The therapeutic effect is determined by; host, viral, and drug factors, and host factors include; age, gender, severity of hepatic fibrosis, fatty liver changes, and insulin resistance. In 2009, a study performed to identify single nucleotide polymorphisms (SNPs) that determine the effect of IFN-based therapy in chronic hepatitis C patients revealed the importance of a SNP near the IL28B gene [70]. IL28B (also called IFNλ3) is thought to induce antiviral activity via the JAK-STAT pathway through phosphorylation of STAT1/STAT2 and the subsequent induction of the IFN stimulating gene (ISG) [71]. In addition to the genotype and viral load of HCV RNA, mutations of amino acids in the core [72] and the interferon sensitivity-determining region (ISDR) [73] have been reported as viral factors that determine the effect of IFN-based therapy. In multivariate analysis of host and viral factors, a high platelet count with mild hepatic fibrosis (F0-1), ≥ 2 ISDR mutations, and the IL28B SNP as the major allele were identified as factors associated with SVR [74]. A lower γGTP level, and milder inflammation, fibrosis, and fatty changes were found in patients with the IL-28B SNP as the major allele, compared to those with this SNP as the minor allele [74]. This suggests that the IL-28B SNP is involved in the progression of hepatitis C pathology, as well as in the therapeutic effect. These results indicate that SVR is likely in PNALT cases. However, there has been no report of an IL-28B SNP associated with PNALT. Drug factors that influence the therapeutic effect include; the type of IFN and the dose and duration of administration of IFN or ribavirin. Novel drugs, such as protease and polymerase inhibitors, will soon become available. In combination therapy with PEG-IFN, ribavirin and protease inhibitor, the overall SVR rate should increase to more than 60% in patients with genotype 1b and a high viral load. In addition, SVR was achieved in 84% of cases with IL-28B SNP as the major allele and glutamine or histidine at position 70 in the core protein, and in 50% of cases with IL-28B SNP as the minor allele and arginine at position 70 [75]. These viral factors and host factors require further investigation in HCV carriers with PNALT.

### 3.2. Antiviral Treatment in HCV Carriers With PNALT

The treatment of chronic hepatitis C has improved significantly over the last few years. Detailed analysis of host factors including the IL28B SNP and viral factors may improve the accuracy of predicting IFN-based therapy effects. For example, this may be based on a prediction of the therapeutic effects using data mining of the blood test results [74][76]. Establishment of tailor-made therapies may be possible with further investigation of SNPs and other data for prediction of IFN-based treatments, including those for PNALT cases. Antiviral therapy for PNALT cases has been estimated to reduce the prevalence of liver cirrhosis and complications by 22% and 16%, respectively, and to decrease the mortality rate by 14% [77]. The AASLD practice guidelines suggest that a decision to use PEG-IFN plus ribavirin therapy should be made based on; a histological evaluation, the possibility of adverse effects, prediction of therapeutic effects, and the presence of complications, regardless of the ALT level [9]. In addition, Okanoue et al. reported that the combination of ALT and platelet counts is useful for evaluating the fibrosis stage in HCV carriers with normal serum ALT levels, and that most patients with platelet counts < 150000/μL are candidates for antiviral therapy, especially those with ALT levels ≥ 31 U/L when the focus is placed on the inhibition of HCC development [27]. Puoti et al. have also found that a rapid virological response was a predictor of sustained response in HCV carriers with PNALT [69]. Thus, although further studies are required to determine whether antiviral therapy should be given in all PNALT cases, it seems likely that many HCV carriers with PNALT should be treated with IFN-based therapy. Tailor-made therapies may also be possible based on the accumulation of data for the prediction of IFN-based treatment efficacy in PNALT cases.

## 4. Conclusion

In this review, we have described the clinical characteristics of HCV carriers with PNALT. For HCV carriers, it is important to minimize complications such as liver cirrhosis and HCC through careful observation and treatment at the appropriate time. For PNALT cases, the course and prognosis should also be monitored and treatment should be considered at an early stage.
